# Modeling the adsorption of hydrogen, sodium, chloride and phthalate on goethite using a strict charge-neutral ion-exchange theory

**DOI:** 10.1371/journal.pone.0176743

**Published:** 2017-05-02

**Authors:** Cristian P. Schulthess, Udonna Ndu

**Affiliations:** 1 Department of Plant Science and Landscape Architecture, University of Connecticut, Storrs, Connecticut, United States of America; 2 Civil and Environmental Engineering Department, Duke University, Durham, North Carolina, United States of America; Brandeis University, UNITED STATES

## Abstract

Simultaneous adsorption modeling of four ions was predicted with a strict net charge-neutral ion-exchange theory and its corresponding equilibrium and mass balance equations. An important key to the success of this approach was the proper collection of all the data, particularly the proton adsorption data, and the inclusion of variable concentrations of conjugate ions from the experimental pH adjustments. Using IExFit software, the ion-exchange model used here predicted the competitive retention of several ions on goethite by assuming that the co-adsorption or desorption of all ions occurred in the correct stoichiometries needed to maintain electroneutrality. This approach also revealed that the retention strength of Cl^−^ ions on goethite increases in the presence of phthalate ions. That is, an anion-anion enhancement effect was observed. The retention of Cl^−^ ions was much weaker than phthalate ions, and this also resulted in a higher sensitivity of the Cl^−^ ions toward minor variations in the surface reactivity. The proposed model uses four goethite surface sites. The drop in retention of phthalate ions at low pH was fully described here as resulting from competitive Cl^−^ reactions, which were introduced in increasing concentrations into the matrix as the conjugate base to the acid added to lower the pH.

## Introduction

The fate and behavior of ions in soil solutions are strongly influenced by their retention on the soil’s solid surfaces. Our best descriptions of these solid-liquid interface reactions make use of adsorption models, and the mathematical predictions generated by these models further allow us to quantify their adsorption strengths. Two additional items gained from adsorption modeling are the total number of solid-phase adsorption sites and the stoichiometry of the reaction involved. It can also elucidate if the ions are competing with each other, or enhancing each other. These bits of information about the retention of aqueous ions on solid surface sites are best described with wet-bench adsorption data (such as data from adsorption isotherms and adsorption envelopes) and adsorption modeling of the data collected.

Thus, adsorption modeling plays a critical role in our understanding of how ions behave in soil environments. There are many types of mechanistic models used to describe adsorption in soils, including single adsorption reactions on single surface sites (such as Langmuir adsorption models [1]), single adsorption reactions on single surface sites with neighboring ion effects on the equilibrium adsorption constants (such as Temkin adsorption models [2]), and single ion-exchange reactions on single surface sites (such as Kerr’s or Gapon’s models [3–5]).

The common denominator in all of these early models is the use of a single reaction on a single surface site. By definition, ion-exchange models involve a competitive reaction, while ion adsorption models do not, at least not by themselves. But note that multiple ion adsorption reactions on a single site will also result in a competitive ion adsorption model. A simple demonstration of this is the pH-competitive adsorption model adapted from enzyme kinetics [6,7]. Modern variations of competitive ion adsorption models involve the presence of surface charge, namely the inclusion of adsorption reactions that change the surface potential. These were introduced by Stumm et al. [8], and excellent modern variations of this type of models are known as charge distribution models [9].

More recently, models have been proposed that involve many adsorption sites. This can range from the simple two-site Langmuir model [10], to the more complex multi-site adsorption reactions involving potential determining ions (also known as MUSIC models [11]). However, strict ion-exchange models involving multiple competitive ions and multiple sites have not been pursued. A strict ion-exchange model assumes that all of the reactions present can only be ion-exchange reactions (that is, none can be simple ion adsorption reactions). An ion-exchange model assumes that the reaction does not affect the surface charge because the adsorption of ions to the surface results either in desorption of an equivalent amount of ions of similar charge from the surface, or the coadsorption of an equivalent amount of ions of opposite charge. Note that ion-exchange models can also include coadsorption reactions.

As with many other modeling approaches, strict ion-exchange models can include multiple adsorption sites and several competing ions, which certainly increases their complexity. However, strict ion-exchange models are much easier to work with overall because they will only involve simple equilibrium constants for each of the proposed equations with no need for intrinsic constants and their corresponding diffuse layer calculations. Their mass balance equations are also easier to solve because they do not include charged surface species, such as SOH_2_^+^ or SO^−^. The question remains, however, do strict ion-exchange models work? If they fail to predict the observed data, then perhaps this approach needs to be abandoned. But if they do predict the data, then, based on Ockham’s Razor, we have a very powerful new tool to work with.

This article seeks to develop an ion-exchange model for the retention of phthalate on goethite. Proper model development will lead to a better understanding of how ions in the soil solution interact with the solid phase. Goethite was selected because it is ubiquitous in soils. It is an iron oxy-hydroxide and its crystallography has been extensively characterized in the literature [12]. Ortho-phthalic acid is also a naturally occurring compound. There are two acid dissociation constants (p*K*a) associated with phthalic acid’s two carboxylic groups: 2.95 [13] and 4.9 [14]. In the environment, phthalic acid is a product of the microbial degradation of phthalic acid esters [15]. Phthalic acid esters are ubiquitous in the environment due to their extensive industrial applications as additives to plastics, paints, and pesticides [16]. High phthalate levels also interfere with normal hormone functions in mammals [17], cause hepatocellular carcinomas in rats [18], and reduce sperm count in men [19].

## Materials and methods

The goethite was synthesized as described by Schwertmann and Cornell [20]. This procedure had previously been confirmed with X-ray diffraction [21] and Fourier transformed infrared and Raman spectra [22] to produce goethite. The goethite surface area was 29.50 m^2^ g^–1^, which was obtained with an accelerated surface area and porosimetry analyzer (Micromeritics, Norcross, GA). The goethite was suspended in water (24 g L^–1^) and was purged continuously with CO_2_ free air to remove any inorganic C contaminants from the solid surface.

The adsorption data used in the ion exchange models described in this study were previously published [23] and are briefly summarized here. The adsorption envelope data used in this study were obtained from two distinct experiments. In the first experiment, a 5-mL aliquot of the goethite suspension was exposed to different quantities of either HCl (0.020 *M*) or NaOH (0.024 *M*) to adjust pH from 2 to 11, and a fixed concentration of NaCl (0.15 m*M*) as an initial background electrolyte. The total volume of each sample was 35 mL, and the goethite concentration in each sample was 3.43 g L^−1^. Batch samples were mixed in a hematology mixer at constant temperature (20°C) for 18–20 hours, then centrifuged to separate the solids from the supernatant solution, and the pH of a portion of the supernatant solution was measured. Other portions of the supernatant were used to assess the amount of H^+^ and Cl^−^ ions that had adsorbed onto the goethite surface. The amount of protons adsorbed were obtained by the backtitration technique [24], while the amount of Cl^−^ adsorbed was quantified by titration of a fixed amount of supernatant with silver nitrate using a Cl^−^ ion selective electrode (Denver Instrument, Denver, CO) according to Midgley and Torrance [25]. The amount of Cl^−^ adsorbed was calculated by subtracting the amount of Cl^−^ remaining in solution from what was added. The second experiment was identical to the first experiment with the exception that 5 mL of phthalic acid (7 m*M*) were added to the goethite suspension in addition to a constant NaCl background electrolyte, and either NaOH or HCl that were used to adjust pH.

The mathematical predictions of the amount adsorbed were generated using IExFit software (version 3.1, distributed by www.alfisol.com). This software was specifically coded to interpret the goodness-of-fit of strict ion-exchange models and to optimize their corresponding equilibrium constants and maximum number of sites. A very important quality of this software is that it allows easy entry of variable initial concentrations of ions as a function of pH. As shall be seen below, this is an essential component for the success of the models proposed. IExFit will also model the aqueous phase speciation of ions, which can influence the affinity of these ions for the solid surfaces present.

## Modeling formulation overview

In order to construct a strict ion-exchange model that is robust enough to be used over a wide range of pH conditions and in the presence of other variables, specific attention must be given to how the reactions are expressed, what is the known or assumed stoichiometry of the reactants involved, what are the total number of surface sites present, and what affinity does each surface have for the ions. Also, attention must be paid to the presence of competitors. Details are presented here to show how each of these items were incorporated into our ion-exchange model.

### Inner-sphere versus outer-sphere adsorption

Ion-exchange models are not able to discern if the adsorbed ion is held as an inner-sphere or outer-sphere ion, nor can they discern if it is held as a mononuclear or binuclear complex. This is because the corresponding mathematics of the models, namely the mass balance and chemical equilibrium equations, are essentially identical regardless of how this detail on the retention of the ion is expressed. The same mathematical results are obtained when expressing the phthalate ligand-surface complex as an inner-sphere (S_2_L) or an outer-sphere ((SOH_2_)_2_L) complex. The actual surface complex can in fact include a combination of the two. Thus, when using the S_2_L or (SOH_2_)_2_L expression in a model, it is often not necessarily suggesting that the surface complex must be an inner-sphere or outer-sphere complex.

Using infrared spectroscopy, Boily et al. [26] reported the existence of a bidentate binuclear outer-sphere complex for phthalate adsorption in the pH range of 3 to 9 and a bidentate mononuclear inner-sphere complex at pH values below 6 on goethite. That is, the retention of these ions is partially both inner and outer sphere, and the strength of retention is a direct result of the degree of inner-sphere retention versus the degree of outer-sphere retention. This dual retention mechanism is commonly observed with adsorbed surface complexes, and the corresponding adsorption strengths of these various surface complexes are not a set of either weak or strong options, but rather a wide gradation of weak to strong options. Mathematically, the wide range of adsorption strengths is expressed as a wide range of adsorption equilibrium values (*K*), where the Gibbs free energy of adsorption (Δ*G*°) is

ΔG°=−RTlnK=ΔH°−TΔS° (1)

where *R* = universal gas constant, *T* = absolute temperature, *ΔH*° = enthalpy, and *ΔS*° = entropy.

The retention of strongly held ions are generally described as inner-sphere adsorption mechanisms. The retention of weakly held ions, such as Cl^−^ and Na^+^, are more difficult to describe. The species descriptions in the mass balance equations used for strict ion-exchange models, such as SOH_2_^+^Cl^−^ and SO^−^Na^+^, suggest that these ions are located in discrete locations controlled by their hydration strengths and surface affinities, which agrees with molecular dynamics simulations [27]. Furthermore, if the counter ion’s location(s) is controlled by its hydration strength and surface affinity [27], then this is equivalent to saying that it is controlled by its adsorption equilibrium constant (*K* = products/reactants). The Gibbs free energy of adsorption quantifies these strengths and affinities, and not their location (namely, inner versus outer sphere).

Thus, the details of retention of ions are not definitively known from their corresponding *K* values. The retention of Cl^−^ anions and Na^+^ cations are known to be weak, and are thus believed to be held by outer-sphere mechanisms. But some exceptions do exist, as was shown with nuclear magnetic resonance spectroscopy by Ferreira et al. [28], where Na^+^ cations are held strongly by an inner-sphere mechanism inside small zeolite channels. The adsorption of these ions on goethite will be expressed here as SONa and SOH_2_Cl, but both are believed to be outer-sphere surface complexes. Furthermore, when stating here that the “surface is protonated”, it does not mean that the counter ion is missing. That is, we speak of SOH_2_^+^Cl^−^(or SOH_2_Cl for short), for example, rather than SOH_2_^+^.

### Monovalent modeling options

Adsorption envelopes display the amount adsorbed as a function of pH. The stoichiometry of reaction is also obtained from these data by comparing the amount adsorbed of each of the components believed present in the adsorption reaction. Ndu and Schulthess [23] observed a nearly equivalent amount of H^+^ cations adsorbing for every Cl^−^ anion adsorbing. This 1:1 stoichiometry for H:Cl adsorption can be expressed as the coadsorption of these ions:

$$\text{SOH }+{\text{ H}}^{+}\;+{\text{ Cl}}^{-}\;⇌{\text{ SOH}}_{2}\text{Cl}$$ (2)

or as release of OH^−^ anions:

$$\text{SOH }+{\text{ H}}_{2}\text{O }+{\text{ Cl}}^{-}\;⇌{\text{ SOH}}_{2}\text{Cl }+{\text{ OH}}^{-}$$ (3)

Since the concentration of H^+^ cations and OH^−^ anions are directly fixed by the pH of the solution, the model predictions resulting from each of these equations are identical. Naturally, however, the corresponding p*K* values for Eqs (2) and (3) will differ by p*K*_w_.

Similarly, we assert here that the adsorption stoichiometry for H^+^ and Na^+^ cations are 1:1, but this was not confirmed for goethite:

$$\text{SOH }+{\text{ Na}}^{+}\;⇌\text{ SONa }+{\text{ H}}^{+}$$ (4)

Once again, Eq (4) predicts identical results as Eq (5) below, but with the difference in their corresponding p*K* values equal to p*K*_w_:

$$\text{SOH }+{\text{ OH}}^{-}\;+{\text{ Na}}^{+}\;⇌\text{ SONa }+{\text{ H}}_{2}\text{O}$$ (5)

The point being made here is that these small variations in how the reaction is actually displayed do not change the model predictions of how much Na^+^ and Cl^−^ adsorb on goethite as a function of pH.

### Divalent modeling options

In sharp contrast to monovalent adsorption processes, the divalent modeling results will differ significantly with variations in how the reactions are displayed. Ndu and Schulthess [23] observed a 2:1 stoichiometry for H^+^ and phthalate (L^2–^) adsorption on goethite. There are different adsorption reaction schemes that one can choose to model this reaction, where each can yield different predictions for adsorption as a function of pH.

**Fig 1:**
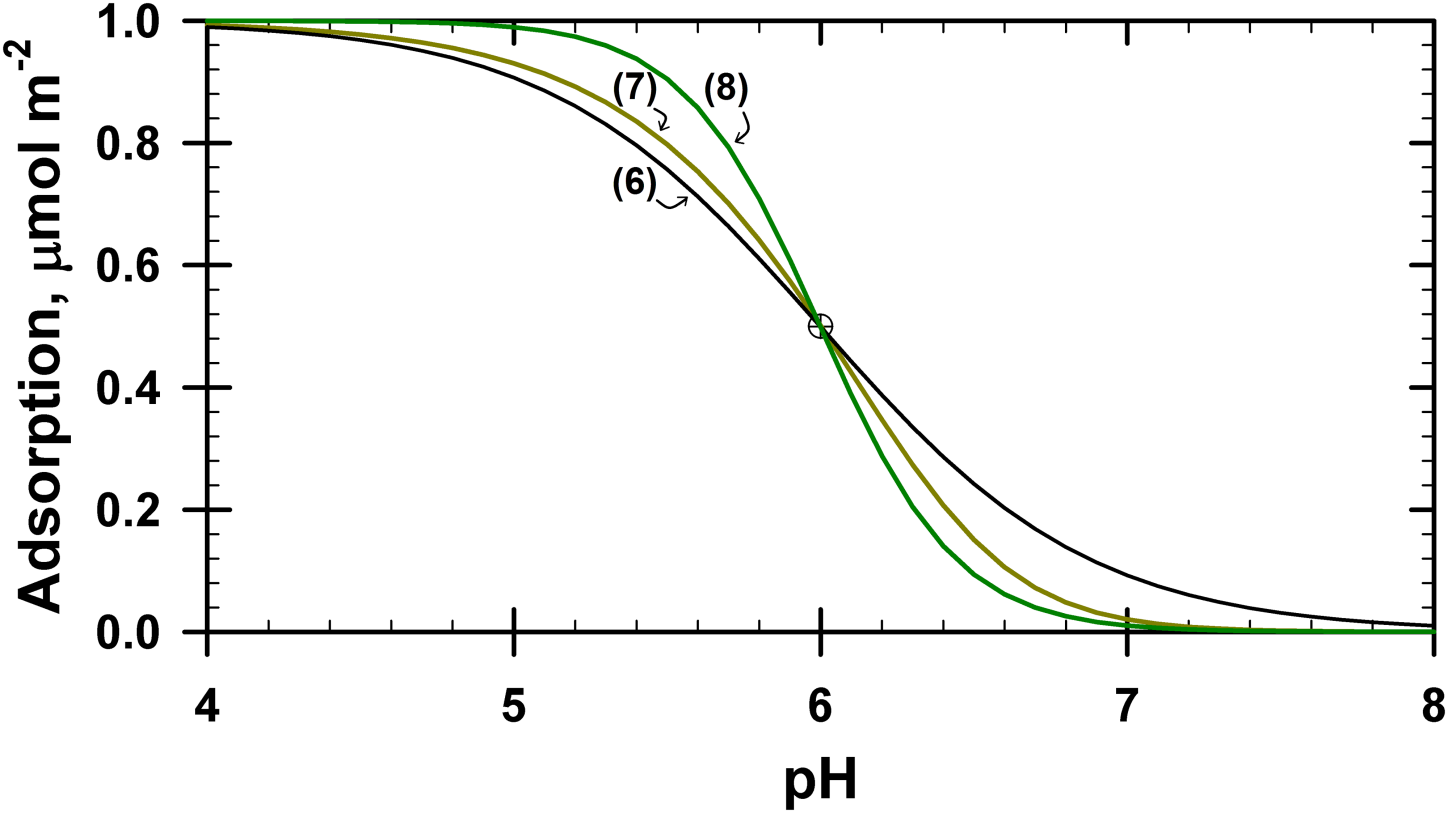
Comparison of predicted results for Eqs (6), (7) and (8). Total phthalate concentration present = 1.0 mmol L^–1^; solid phase adsorption maximum = 2.0 μmol m^–2^ (equivalent to 0.2 mmol L^–1^) for Eqs (6) and (7), or 1.0 μmol m^–2^ (equivalent to 0.1 mmol L^–1^) for Eq (8); p*K*_6_ = –7.51, p*K*_7_ = –18.72 and p*K*_8_ = –15.02. The curves shown and the corresponding p*K* values were optimized using IExFit to fit a single datum point at pH = 6 and phthalate adsorption = 0.5 μmol m^–2^. No aqueous phase protonation reactions were included in this simulation.

The following three equations for phthalate adsorption are very similar, but there are sufficient differences between them so as to yield very different predictions (Fig 1):

$$\text{SOH }+{\text{ H}}^{+}\;+\;\frac{1}{2}{\text{L}}^{2-}\;⇌{\text{ SOH}}_{2}{\text{L}}_{0.5}$$ (6)

versus

$$2\text{SOH }+\;2{\text{H}}^{+}\;+{\text{ L}}^{2-}\;⇌\;{\left({\text{SOH}}_{2}\right)}_{2}\text{L}$$ (7)

versus

$$\text{SOH }+\;2{\text{H}}^{+}\;+{\text{ L}}^{2-}\;⇌{\text{ SOH}}_{2}\text{LH}$$ (8)

Note that *K*_7_ does not equal *K*_6_^2^. Although the stoichiometry of H:L is 2:1 in all these equations, the stoichiometry of the initial surface site and the product surface is 1:1 in Eqs (6) and (8) but 2:1 in Eq (7). Accordingly, this small detail in the formulation of the model directly impacts the stoichiometry of the reactants and products, which in turn influences the resultant curve predictions. For additional clarity, note the subtle differences in the following equation with Eq (6):

$$\text{SOH }+{\text{ H}}^{+}\;+\;\frac{1}{2}{\text{L}}^{2-}\;⇌\;\frac{1}{2}{\left({\text{SOH}}_{2}\right)}_{2}\text{L}$$ (9)

Eq (9) is simply half of Eq (7), and this leads to *K*_7_ = *K*_9_^2^ with an identical curve result for the models based on Eqs. (9) or (7) in Fig 1.

The shape of the predictions generated by Eq (8) is also very different (Fig 1), where the adsorption edge has a very sharp rise. If the monodentate product in Eq (8) occupies two sites, either by its size or by intermittent H-bonding with the neighboring SOH site, then the reaction would be better represented by Eq (7). Thus, knowing that the ion can form a monodentate surface product does not automatically mean that Eq (8) is the preferred reaction to use in the model. We studied all three variations for phthalate ion adsorption, but only Eq (7) will be discussed here because Eq (8) did not yield good results, particularly at low pH values. Furthermore, infrared spectroscopy indicates that phthalate adsorption is bidentate [26]. The adsorption modeling results using Eq (6) were similar to Eq (7), but we used Eq (7) in our model presented below because the adsorption edges were sharper and the data fit was better.

All these equations have different stoichiometry values, which result in different *K* values. They also have different mass balance equations, which together with the differences on how the *K* values are defined yield the very different adsorption patterns shown in Fig 1. Numerous additional output variations can be generated if surface impurities are also believed to be present, which is a particularly sensitive issue when trying to describe or predict the adsorption of weakly adsorbing ions.

In summary, one must decide how to describe the exchange reaction. While the details of how the initial surface site or final surface product are expressed do not influence the results (such as, inner-sphere versus outer-sphere variations), the details of the stoichiometry of the reactants and products do matter greatly. The best way to quantify this for pH-dependent reactions is to compare the amount of components adsorbed with the amount of protons adsorbed over a range of pH values. The collection of proper proton adsorption data is essential for this step, and these are typically also the most difficult data to collect. Proper proton adsorption data require the backtitration technique [24], while ion adsorption data require simple, straight forward final minus initial concentration measurements.

Similar discussions were once common in the literature about how to formulate ion-exchange reactions for monovalent-divalent exchange. A variation to Eq (6) for exchange of cations was first introduced by Gapon [5], and was subsequently applied by Beckett [29] to the derivation of the now well known quantity/intensity curves (Q/I curves) for predicting the supply of nutrients to plants. Current use of equations similar to Eq (6) outside of Q/I curves is rare, particularly for anions. Eqs (7) and (8) are commonly applied to modern models, and were probably first introduced or popularized by Kerr [3, 4] for cation ion-exchange models.

Predictions based on Eqs (6)–(8) often failed, and so early researchers also often discussed how to properly express the corresponding equilibrium constants [30–33]. That is, they also discussed alternatives to the well established equilibrium equations of products over reactants in molar units for the proposed adsorption reactions. Schulthess and Sparks [34] noted that these early publications on the subject were entrenched in describing these ion-exchange reactions as simple single reactions rather than multiple competitive reactions or multisite reactions. For example, Posner and Bowden [35] challenged some of the early efforts to develop multisite models. However, if the adsorption process involves multiple surfaces and/or multiple pH-dependent competing ions, then there is no single reaction that can properly mimic these various reactions and no single equilibrium constant that will equal these various competing equilibria. Thomas [36] even suggested that we should consider using an equilibrium coefficient rather than an equilibrium constant. Clearly, there was much frustration with how to make these models work at the time. Thus, the inclusion of competitors (which can vary in concentration as a function of pH) and multiple adsorption sites (which vary in reactivity) are discussed below because they are essential for the development of robust ion-exchange models.

### Impact of competitors

**Fig 2:**
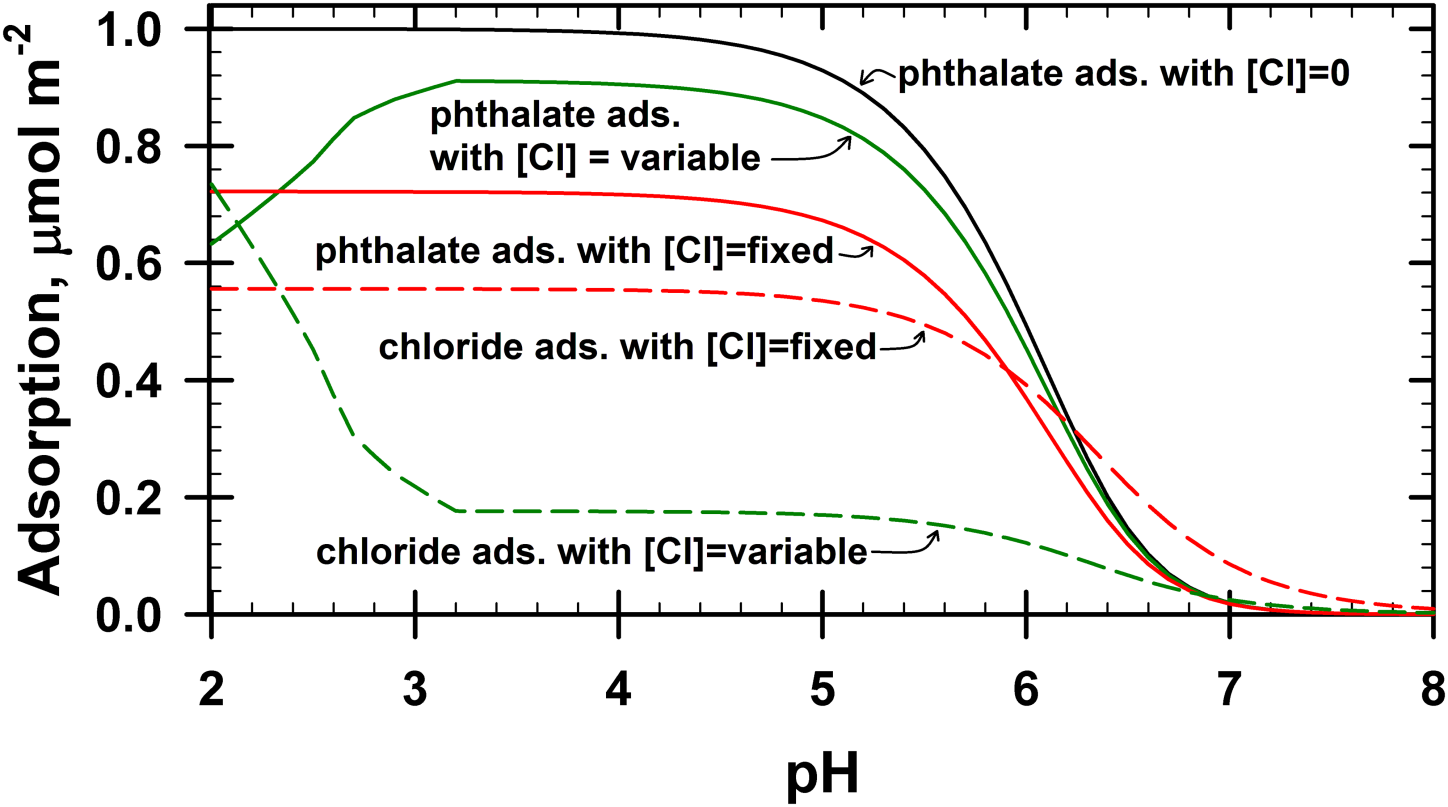
Comparison of predicted results for Eqs (2) and (7). Total phthalate concentration present = 1.0 mmol L^–1^, solid phase adsorption maximum = 2.0 μmol m^–2^ (equivalent to 0.2 mmol L^–1^), p*K*_2_ = –8 and p*K*_7_ = –18.7. The initial chloride concentrations were either zero, fixed value (4.54 m*M*), or pH dependent variable values. For the pH dependent variable values, the Cl^−^ values were fixed at 1.27 m*M* above pH 3.18, but increased due to addition of HCl acid below pH 3.18 (total Cl^−^ input at pH 2.33 was 4.54 m*M*). Resultant curves were generated by IExFit. No aqueous phase protonation reactions were included in this simulation.

The total number of adsorption sites will be occupied by any of the available ions competing for these sites. That is, all adsorption sites are always occupied by one of the competing ions. Accordingly, if we have phthalate and Cl^−^ in competition with each other for adsorption on the same SOH sites, such as Eqs (2) and (7), then the resultant adsorption curves will change based on the relative concentration of each of these ions, and on whether the total concentrations present for each of these ions are constant. Fig 2 illustrates this with phthalate and Cl^−^ adsorption, but note that the reaction conditions applied to Fig 2 (namely, *K* values and total number of sites) were arbitrary so that the impact of competing reactions on the shape of the adsorption envelopes is clearly recognized. The phthalate aqueous protonation reactions were ignored here so that only specific components on the impact of Cl^−^ adsorption on phthalate adsorption are illustrated.

In absence of Cl^−^ in Fig 2, the predicted phthalate adsorption increases sharply with each adsorbed molecule occupying two sites and reaching 1.0 μmol m^–2^ adsorption below pH 4. If phthalate aqueous protonation reactions are also included, then the shape of the predicted phthalate adsorption in absence of Cl^−^ would be nearly the same (not shown).

Now, if Cl^−^ ions are present but at a constant (fixed) concentration, then the shape of the amount of phthalate adsorbed is the same as before but it now never reaches 1.0 μmol m^−2^ adsorption. This time, instead, phthalate reaches a maximum of 0.722 μmol m^–2^ adsorption below pH 4, while the Cl^−^ ions occupy the remaining sites with 0.556 μmol m^−2^ adsorption below pH 4. Note that for this simulation (0.722 * 2) + (0.556 * 1) = 2 μmol m^−2^ = total number of sites available. It is also important to note the shape of the phthalate adsorption curve in the presence of a constant Cl^−^ competitor. It is simply lowered but otherwise identical to the curve without the presence of Cl^−^ ions. The lowering of the phthalate adsorption curve also gives the illusion that the adsorption edge has moved left toward lower pH values; but it has not. It has only lowered vertically. The lowering of this adsorption curve is not due to a compression of some diffuse double layer near the solid-liquid interface, but rather to the competition by other anions for the same adsorption sites.

In the presence of a variable concentration of Cl^−^ ions, the shape of both the phthalate and Cl^−^ adsorption curves change significantly. The concentration of Cl^−^ will change if the acid used to adjust the pH is HCl. It is impossible to add a strong acid without also generating its conjugate base. In the simulation shown in Fig 2, the initial NaCl concentration was low, but the Cl^−^ concentrations increased below pH 3.18. Notice in this simulation that as the Cl^−^ concentrations started to increase below pH 3.18, the phthalate adsorption decreased and the Cl^−^ adsorption increased. That is, the drop in phthalate adsorption is not due to any change in the solid’s surface charge or surface protonation, but simply to the presence of more Cl^−^ ions eager to behave competitively. It is very common to ignore the role of the conjugate base in adsorption modeling, and this is probably because the adsorption of Cl^−^ ions are perceived as being too weak to be significantly competitive. This is true, but even weakly held ions will be significantly competitive when their concentrations are increased. This detail is critical and should not be ignored. The IExFit software was specifically coded to allow inclusion of these conjugate ions (namely, the concentrations added as a function of equilibrium pH).

The Na^+^ concentrations also increased above pH 3.18 in this simulation due to the addition of NaOH. It is impossible to add a strong base without also generating its conjugate acid. However, there were no competitive Na^+^ adsorption reactions added to the simulation shown in Fig 2. If competitive Na^+^ adsorption reactions were added to this simulation, then phthalate’s adsorption edge and/or shape of the edge could change slightly, depending on the p*K* values used in the simulation for sodium’s adsorption reaction. The phthalate adsorption edge could appear pushed slightly toward lower pH values, and the shape of the edge would decrease more sharply on the higher pH side of the edge.

### Number of different adsorption sites

A fixed total number of adsorption sites is a key feature of mechanistic adsorption models. The Langmuir equation [1] is a classical example of this, which assumes one reaction with a specific 1:1 stoichiometry and fixed energy of adsorption. Another is the Tempkin equation [2], which involves a single adsorption reaction with adsorbate-adsorbate interactions resulting in a linear decrease in adsorption strengths with coverage. The Freundlich equation is not a mechanistic model, but it can also be derived by assuming that the free surface energy is linearly related to the fraction of surface coverage [37]. There is also the Dubinin—Radushkevich equation, which assumes a single adsorption reaction with a Gaussian adsorption energy distribution of a heterogeneous surface [38]. All of these are adsorption reactions and not exchange reactions. Since they lack competitive ions, they are not able to include pH (H^+^ and OH^−^ ions) or other ions as competing components, which greatly reduces their robustness. But most limiting for these models is their inability to specifically identify what the adsorbing surfaces are—and in soils there are obviously more than one. Even single minerals will have more than one, as noted below. Conversely, modern surface complexation models will often include adsorption competition and multiple surfaces.

The total number of adsorption sites is relatively easy to extract from adsorption data. This is generally equivalent to the maximum amount of adsorption observed in absence of competition, or to the numerical sum of all the adsorbing species in the presence of competition. The number of different adsorption sites is more difficult to deduce. In broad terms, the number of different adsorption edges will equal the minimum number of different adsorption sites. But this may not always work well because technically the number of different adsorption edges equals the minimum number of different surface reactions, and the assertion that different surface reactions are due to different adsorption sites is not always true. It is therefore helpful to have some independent estimate of the number of different adsorption sites expected on the solid phase.

**Fig 3:**
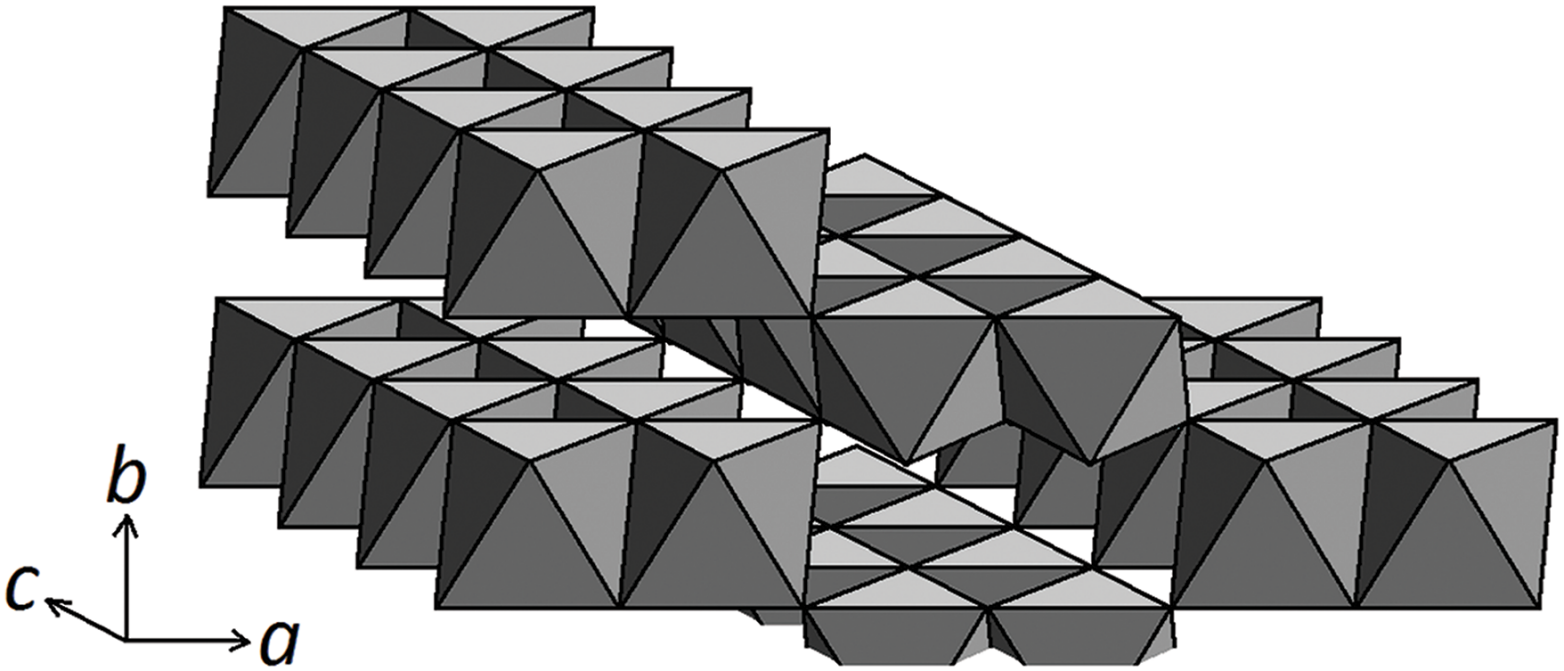
Structure of goethite, α-FeOOH. The double chains of octahedra run parallel to *c*. Note four possible adsorption sites on the {110} face consisting of singly, doubly and triply coordinated hydroxyl groups, plus a fourth consisting of two hydroxyl groups on one Fe atom.

Fig 3 shows the crystal structure of goethite, where the Fe atoms are at the center of each octahedra. The dominant face in natural and synthetic goethite is {110}, but it will also have {100}, {021} and {010} faces [39]. The O and OH atoms are at the octahedral corners. Unlike dry solid samples, in aqueous suspensions most of the surface O atoms will be protonated, forming surface -OH groups, and these -OH groups will be coordinated with one, two or three Fe atoms, depending on their location in the structure. Each of these FeOH surface groups are potential adsorption sites for ions in solution. Based on the Pauling bond valence principle [40], where the charge of an ion is shared with all its surrounding ligands, the reactivity of the surface site is directly related to the coordination number of the -OH group on the site.

Using crystallographic considerations, four types of hydroxyl groups on goethite have been identified based on the number of Fe atoms that these hydroxyl groups coordinate with [41–45]. When a hydroxyl group is bound to one Fe atom, two Fe atoms or three Fe atoms, they are said to be singly, doubly, and triply coordinated, respectively. However, a fourth coordination exists when one Fe atom is bound to two hydroxyl groups. Infrared spectra of iron oxides display several bands, which point to several kinds of surface OH groups [46]. Spectroscopy has shown that there are four types of surface hydroxyl groups on goethite, and these groups have different reactivities [47]. Boily and Kozin [48] state that the densities and distributions of these groups are determined by the orientation of the crystal surface, and that the reactive groups in the goethite crystalline structure are more uniformly distributed than in lepidocrocite. Villalobos and Pérez-Gallegos [44] argue that doubly coordinated groups tend to be unreactive except when present on the {210} and {010} faces of the goethite crystal, while triply coordinated groups are present on faces {101} and {001} but are considered unreactive.

Values for the p*K*a’s of the goethite surface (hydr)oxo groups vary in the literature depending on the method of computation. With the use of the CD-MUSIC model, the p*K*a’s of 4 different surface (hydr)oxo groups of goethite have been calculated [43, 49]. Of these 4 groups, it is thought that only one group is involved in environmental reactions. In the study by Leung and Criscenti [42], the p*K*a of a doubly protonated oxygen atom bonded to a single Fe atom (Fe_I_OH_2_^+^) on goethite’s {101} surface was estimated to be 7, which they obtained with the use of *ab initio* molecular dynamics simulations and potential-of-mean-force techniques.

## Results and discussion

It is reasonable to postulate that the adsorption of weakly held ions is very sensitive to very small changes in the matrix, particularly to the presence of competing ions and surface contaminants. Accordingly, modeling the retention of weakly held ions is inherently more difficult than modeling the retention of strongly held ions. The goethite sample used in this study was purged with N_2_ gas to remove the autochthonous (native) inorganic C species, namely adsorbed HCO_3_^−^ species, which are known to be retained by goethite minerals [50, 51]. Since the goethite was synthesized, no additional competitive ions were known or expected to be present, and therefore no additional precautions were taken to clean the goethite surface.

**Fig 4:**
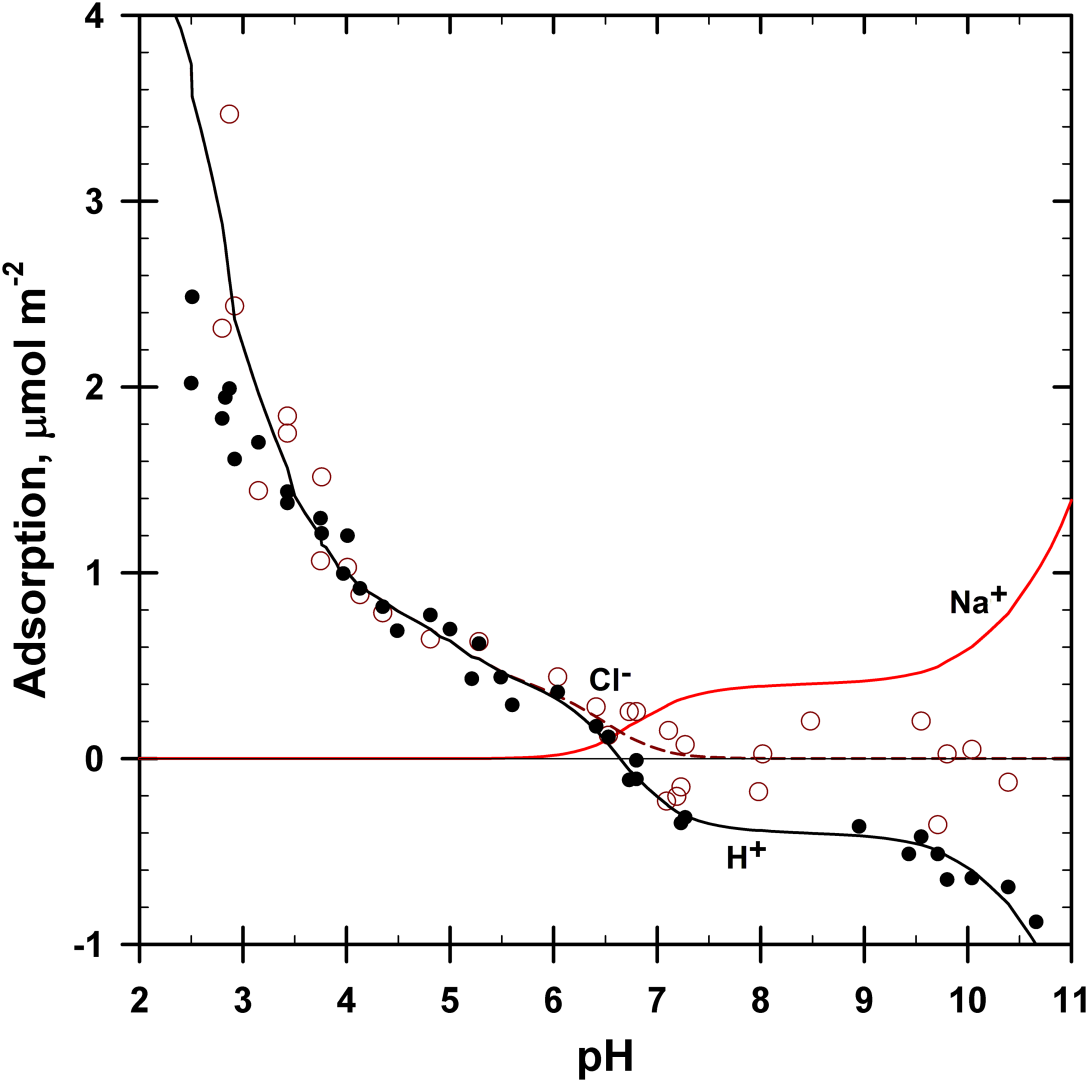
Adsorption of H^+^, Na^+^ and Cl^−^ ions on goethite using the 4-site model described in Table 1. Symbols: solid = H^+^, circles = Cl^−^. The curves shown were generated and optimized using IExFit software.

The adsorption of H^+^ and Cl^−^ ions is shown in Fig 4 (from [23]). The proton data were offset by 0.50 μmol m^−2^ based on the PZSE offset of the backtitration results [23]. The Cl^−^ data were offset by 0.11 μmol m^−2^ based on the desorption values observed at high pH [23]. That is, the goethite had autochthonous Cl^−^ species present that were noticed when they desorbed at high pH. This offset also meant that the total Cl^−^ concentrations present were increased by 0.011 m*M*.

The stoichiometry of H:Cl coadsorption in Fig 4 is 1:1, as was discussed by Ndu and Schulthess [23]. In the pH range of 6.5 to 2.8, each increment in Cl^−^ adsorption on goethite was accompanied by an equivalent amount of H^+^ adsorption. Similar Cl^−^ adsorption results were observed for goethite by Nanzyo and Watanabe [52].

At higher pH (> 6.5), there is H^+^ desorption but no apparent Cl^−^ activity. In keeping with a strict ion-exchange theory, we assume here that the desorption of H^+^ from the goethite mineral surface is accompanied with the concurrent adsorption of Na^+^ cations, which was also previously measured by Nanzyo and Watanabe [52]. The adsorption of Na^+^ cations was not measured in this study. The zero point of titration (ZPT) for this sample was at pH 6.41, from where NaOH was added to raise the pH and HCl was added to lower the pH resulting in higher concentrations of Na^+^ or Cl^−^ ions at pH values far from 6.41.

The data shown in Fig 4 was modeled based on the 4-site ion-exchange model described in Table 1. Four sites were used for reasons discussed above. The total number of sites of 4.4 μmol m^−2^ was set based on proton adsorption data collected in the presence of phthalate, which will be discussed later below. The adsorption max (Г_max_) values for each of the four sites were selected as follows: 0.4 μmol m^–2^ for site Sd was based on the negative proton adsorption observed from pH 9 to 7 and a similar positive Cl^−^ and proton adsorption from pH 6 to 5.5; 0.5 μmol m^−2^ for site Sc was based on the Cl^−^ and proton adsorption values observed near pH 4; 1.3 μmol m^–2^ for site Sb was based on the proton adsorption values observed near pH 3 to 2.5; and 2.2 μmol m^–2^ for site Sa was based on the remaining value needed to reach a total of 4.4 μmol m^–2^.

**Table 1:** Adsorption parameters for retention of Cl^−^ ions on goethite. Results for this four-site model are shown in Fig 4. This ion-exchange model assumes a 4.4 μmol m^–2^ total number of surface site. For each surface site (Sa, Sb, Sc and Sd) the adsorption max = total number of sites = {SOH} + {SOH_2_Cl} + {SONa}.

Ads. Max. μmol m^-2^	*pK*	Ion-Exchange Reaction	
2.2	-5.6	$\text{SaOH }+{\text{ H}}^{+}\;+{\text{ Cl}}^{-}\;⇌{\text{ SaOH}}_{2}\text{Cl}$	(10)
2.2	8.8 (est.)	$\text{SaOH }+{\text{ Na}}^{+}\;⇌\text{ SaONa }+{\text{ H}}^{+}$	(11)
1.3	-6.6	$\text{SbOH }+{\text{ H}}^{+}\;+{\text{ Cl}}^{-}\;⇌{\text{ SbOH}}_{2}\text{Cl}$	(12)
1.3	8.8 (est.)	$\text{SbOH }+{\text{ Na}}^{+}\;⇌\text{ SbONa }+{\text{ H}}^{+}$	(13)
0.5	-8.7	$\text{ScOH }+{\text{ H}}^{+}\;+{\text{ Cl}}^{-}\;⇌{\text{ ScOH}}_{2}\text{Cl}$	(14)
0.5	6.8	$\text{ScOH }+{\text{ Na}}^{+}\;⇌\text{ ScONa }+{\text{ H}}^{+}$	(15)
0.4	-10.5	$\text{SdOH }+{\text{ H}}^{+}\;+{\text{ Cl}}^{-}\;⇌{\text{ SdOH}}_{2}\text{Cl}$	(16)
0.4	2.8	$\text{SdOH }+{\text{ Na}}^{+}\;⇌\text{ SdONa }+{\text{ H}}^{+}$	(17)

The p*K* values for the reactions shown in Table 1 were optimized using IExFit software. The p*K*_16_ and p*K*_17_ values control the model fit near the PZSE and were optimized concurrently because they influence each other, as was discussed earlier in the Modeling Formulation Overview section. Following this, all other p*K* values were optimized in segments. The p*K*_15_ value was optimized to fit the data near pH 10. The p*K*_11_ and p*K*_13_ values were estimated because they predict data above pH 10.7 that were not collected.

The curve fit is very good to excellent for all ions in Fig 4 (R^2^ = 0.92 for Cl^−^ and R^2^ = 0.77 for H^+^; also, R^2^ = 0.99 for the curve fit with H^+^ data above pH 3.5). The higher the number of adjustable parameters, the more likely that a good fit with the data will be achieved. The data displayed four different adsorption maxima, and crystallographic considerations suggest that four different reactive surfaces exist on goethite. Also, adsorption modeling efforts based on two or three surface sites rather than four resulted in poor fits (not shown).

**Fig 5:**
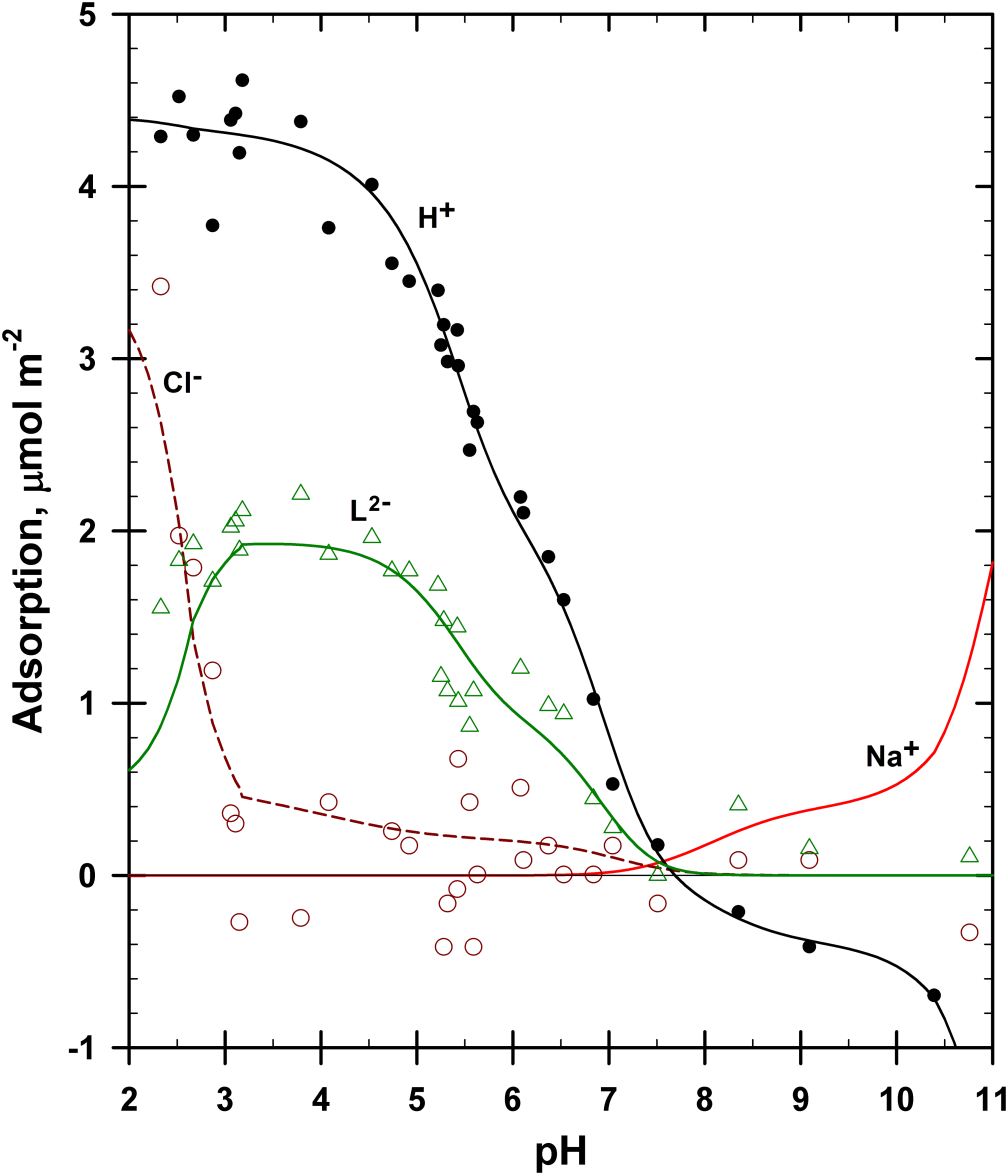
Adsorption of H^+^, Na^+^, Cl^−^ and phthalate (L^2–^) ions on goethite using the competitive ion-exchange model described in Table 2. The aqueous phase protonation reactions of phthalate ions were also included in the model. Symbols: solid = H^+^, circles = Cl^−^, triangles = L^2–^. The curves shown were generated and optimized using IExFit software.

If an ion behaves in the same way with various different sites, then the distinction between these sites disappears. This would result in a model with fewer distinctly different adsorption sites. If ion adsorption is strong, then small differences in surface reactivity among various different surface sites may go unnoticed. This appears to be the case with phthalate adsorption, shown in Fig 5.

The data collected for phthalate adsorption on goethite found the H:L stoichiometry to be 2:1 [23]. The data also suggested that competitive Cl^−^ adsorption was causing the drop in phthalate adsorption at low pH. The evidence for this was two fold. First, an increase in Cl^−^ adsorption was observed at low pH in the same pH range where the decrease in phthalate adsorption was observed. Second, the proton adsorption data that were very precisely collected using the backtitration technique showed no change in values in this low pH range. That is, even while the Cl^−^ adsorption increased and the phthalate adsorption decreased, there was no change in proton adsorption. This stability in the proton adsorption data can only occur if the adsorption of these two anions are on the same sites and in the correct stoichiometric proportions. The Cl^−^ and L^2–^ anions cannot be interacting with the surface independently of each other.

A competitive ion-exchange model is proposed here for describing the phthalate adsorption data collected (Table 2). It is a 4-site model because, as discussed earlier, goethite is known to have four sites. However, a nearly identical 2-site model can be constructed for this data set, with each site having an adsorption maximum of 2.2 μmol m^−2^. The data shown in Fig 5 clearly show two adsorption edges and two adsorption maxima, which makes it very tempting to pursue a much simpler 2-site model instead. That is, sites Sb, Sc and Sd can be combined and treated as one site with a maximum of 2.2 μmol m^−2^. One problem in taking this shortcut is noticed with Eqs. (28) and (29) for site Sd in Table 2 and with the proton desorption data at high pH (>8). If the proton desorption data at high pH are assumed to be correlated in these ion-exchange models with Na^+^ adsorption, then the 0.4 μmol m^−2^ proton desorption values at pH 9 would need to have their own site because the other sites for phthalate have a 2.2 μmol m^−2^ maxima, which is too large, and it would contradict the claim of having a 2-site model. Furthermore, if the Na^+^ adsorption reaction is treated as occurring on a separate adsorption site, then its competition with the adsorption of phthalate ions will be missed and this will result in a different p*K* optimization result for the phthalate ions. The optimization of Eqs. (28) and (29) are for data in the same pH range (6.5 to 9.1), and they thus influence each other. All of this debate occurs because the phthalate ions are adsorbing much more strongly than the Cl^−^ ions, which resulted in several slightly different adsorption sites being well differentiated in the Cl^−^ adsorption envelope but not in the phthalate adsorption envelope.

**Table 2:** Adsorption parameters for retention of phthalate (L^2–^) ions on goethite. Results for this model are shown in Fig 5. This ion-exchange model assumes a 4.4 μmol m^–2^ total number of surface site, with the same adsorption maxima values used in Table 1. For each surface site (Sa, Sb, Sc and Sd) the adsorption max = total number of sites = {SOH} + {SOH_2_Cl} + {SONa} + 2{(SOH_2_)_2_L}.

Ads. Max. μmol m^-2^	*pK*	Ion-Exchange Reaction	
2.2	-6.6	$\text{SaOH }+{\text{ H}}^{+}\;+{\text{ Cl}}^{-}\;⇌{\text{ SaOH}}_{2}\text{Cl}$	(18)
2.2	8.8 (est.)	$\text{SaOH }+{\text{ Na}}^{+}\;⇌\text{ SaONa }+{\text{ H}}^{+}$	(19)
2.2	-17.4	$2\text{SaOH }+\;2{\text{H}}^{+}\;+{\text{ L}}^{2-}\;⇌\;{\left({\text{SaOH}}_{2}\right)}_{2}\text{L}$	(20)
1.3	-6.6	$\text{SbOH }+{\text{ H}}^{+}\;+{\text{ Cl}}^{-}\;⇌{\text{ SbOH}}_{2}\text{Cl}$	(21)
1.3	8.8 (est.)	$\text{SbOH }+{\text{ Na}}^{+}\;⇌\text{ SbONa }+{\text{ H}}^{+}$	(22)
1.3	-20.8	$2\text{SbOH }+\;2{\text{H}}^{+}\;+{\text{ L}}^{2-}\;⇌\;{\left({\text{SbOH}}_{2}\right)}_{2}\text{L}$	(23)
0.5	-8.7	$\text{ScOH }+{\text{ H}}^{+}\;+{\text{ Cl}}^{-}\;⇌{\text{ ScOH}}_{2}\text{Cl}$	(24)
0.5	8.8	$\text{ScOH }+{\text{ Na}}^{+}\;⇌\text{ ScONa }+{\text{ H}}^{+}$	(25)
0.5	-20.8	$2\text{ScOH }+\;2{\text{H}}^{+}\;+{\text{ L}}^{2-}\;⇌\;{\left({\text{ScOH}}_{2}\right)}_{2}\text{L}$	(26)
0.4	-10.5	$\text{SdOH }+{\text{ H}}^{+}\;+{\text{ Cl}}^{-}\;⇌{\text{ SdOH}}_{2}\text{Cl}$	(27)
0.4	5.4	$\text{SdOH }+{\text{ Na}}^{+}\;⇌\text{ SdONa }+{\text{ H}}^{+}$	(28)
0.4	-20.8	$2\text{SdOH }+\;2{\text{H}}^{+}\;+{\text{ L}}^{2-}\;⇌\;{\left({\text{SdOH}}_{2}\right)}_{2}\text{L}$	(29)

Since the four sites are poorly differentiated on the strongly adsorbing phthalate adsorption envelope, a compromise was pursued here whereby three of the p*K* values for the phthalate adsorption reactions in Table 2 (Eqs. (23), (26) and (29)) were treated as one, but the adsorption maxima used in Table 1 were preserved. In other words, it is a pseudo 2-site phthalate adsorption model presented as a 4-site model to maintain consistency with the model described in Table 1. Or stated again differently, it is a 4-site model with the strongly held phthalate ions behaving identically on three of the sites, forcing the entire 4-site model to resemble a 2-site model relative to phthalate. Nevertheless, the basic components of the 4-site model described in Table 1 are maintained in Table 2, and the Γ_max_ values for each of the sites previously identified when using the weakly adsorbing Cl^−^ ion are also maintained. Note that the IExFit software code allows optimization be done this way when requested. That is, any of the p*K* values in different sections of the model (or rows in Table 2) can be optimized or treated as one.

The p*K*_25_ value for Na^+^ adsorption was optimized using the proton adsorption data at pH 10.39. The change in this value from Table 1 will be discussed later below, but for now note that if p*K*_25_ = p*K*_15_ the curve fit would be poor. The p*K*_22_ and p*K*_19_ values are for Na^+^ adsorption at higher pH values, and were simply set equal to p*K*_25_. As in Table 1, they are also labeled as estimated values in Table 2. As noted above, p*K*_28_ for Na^+^ adsorption and p*K*_29,26,23_ for phthalate adsorption were optimized concurrently for the pH data range 6.0 to 10. Once again note the change in p*K*_28_ with the p*K*_17_ value for Na^+^ adsorption. This will also be discussed later below, but again note for now that if these are equal the curve fit would be poor.

The p*K*_27,24,21_ values for Cl^−^ adsorption are the same values as those determined earlier in Table 1. Next, the p*K*_20_ value for phthalate adsorption was optimized using the data below pH 6. Finally, the p*K*_18_ value for Cl^−^ adsorption was optimized for the low pH values, which differs from p*K*_10_ in Table 1 and will be discussed later below; the curve fit would be poor for Cl^−^ adsorption if these were the same.

With few exceptions as noted above, the p*K* values in Table 1 were repeated in Table 2. The optimization of all of these p*K* values was based on the best curve fit with the adsorption data. Note that the concurrent protonation reactions of phthalate ions in the aqueous phase influences the adsorption p*K* values, and are therefore included in the model. The p*K* value for the formation of the Na-phthalate ion-pair is –0.7 [53], which was also included in the model via the IExFit user defined database. The calculated Na-phthalate ion-pair concentrations accounted for less than 1% of the total aqueous phthalate concentrations for all conditions below pH 10.5.

In absence of a competitive Cl^−^ adsorption reaction, the model will not show any change in phthalate adsorption at low pH values. This is similar to what was illustrated earlier in Fig 2. However, in our competitive ion-exchange model, the increase in Cl^−^ adsorption is causing the decrease in phthalate adsorption at low pH values, and the competition is net charge-neutral and stoichiometrically balanced. That is, the Cl^−^ ions and the phthalate ions are competing for the same sites. This competition is a key component of the model described in Table 2, and the fact that the resultant model predictions also show this competition at low pH validates and quantifies this competitive behavior. Note also that the predicted Cl^−^ adsorption is greatly amplified by the phthalate protonation reactions in the aqueous phase, and thus the inclusion of these reactions is important for proper optimization of the parameters.

In Fig 5, the retention of Cl^−^ ions is nearly zero in the presence of phthalate for all pH values above pH 3.2, but have a sharp increase in Cl^−^ retention at low pH (< 3.2). The ZPT for this mixture was pH 3.18. This increase in Cl^−^ retention coincides with the addition of HCl to lower the pH below 3.18, which resulted in a concurrent increase in the conjugate base concentrations, namely the total Cl^−^ anion concentrations. Although weak, Cl^−^ ions are competitive at higher concentrations. The important advantage of using IExFit software for this competitive ion-exchange model is that the actual Cl^−^ concentrations added to the matrix as a function of pH can be included in the code’s mass balance calculations. The competitive Cl^−^ ions are added whenever HCl solutions are added to lower the pH.

The H_2_L species do form in solution (p*K*a_1,2_ = 2.95 and 4.9; [13, 14]), and these reactions were included in the model optimization. The IExFit software will also generate speciation diagrams for each of the aqueous species present (H_2_L, HL^−^, L^2−^ concentrations versus pH), which showed 81% phthalate ions remaining in solution at its lowest at pH 3.5. Note that the protonated species in solution should be easier to adsorb on goethite if the L^2−^ adsorption reaction involves the coadsorption of two H^+^ ions, as the model presented in Table 2 suggests. That is, the adsorption of HL^−^ plus one H^+^ should be easier than the adsorption of L^2−^ plus two H^+^. The former has fewer species that must concurrently find their way to the surface site, and therefore will be easier to adsorb. That is, the entropy change (Δ*S*°) should favor the reaction with fewer species involved. Similarly, the adsorption of H_2_L should be easier than the adsorption of HL^−^ plus one H^+^. Accordingly, the increase in phthalate adsorption at around pH 5 coincides with phthalate’s p*K*a value.

The curve fit is very good to excellent for all ions in Fig 5 (R^2^ = 0.82 for phthalate, R^2^ = 0.80 for Cl^−^, R^2^ = 0.99 for H^+^). The Cl^−^ adsorption data had a great deal of variability and were difficult to quantify properly with the ion selective electrode and ion titration method. Conversely, the phthalate data were easy to collect with the total organic analyzer, and the proton data were easy to collect with the backtitration technique. Our ability to simultaneously model all these diverse ions in a single sweep was strongly dependent on our ability to properly collect all of the needed data. This is particularly true with the proton adsorption data, which were needed to collect the stoichiometry information for each of the reactions. The proton adsorption data were also used to quantify the total number of surface sites, and to verify that changing the retention of the competing Cl^−^ ions at low pH did not impact the total proton adsorption values.

Comparing the optimization results in Tables 1 and 2, we see a large change in the p*K* value for the weakest Cl^−^ ion-exchange reaction shown on the Sa surface sites (Eqs. (10) and (18)). Although initially very weak, the value increased in Cl^−^ adsorption strength from –5.6 for p*K*_10_ to –6.6 for p*K*_18_. The increase in Cl^−^ adsorption strength described in Table 2 is probably a result of the adsorption of phthalate ions. This seems counterintuitive because L^2−^ and Cl^−^ are competing with each other for surface sites, but similar anion-anion enhancement interactions have been observed before. Wijnja and Schulthess [51] observed enhanced adsorption of SeO_4_^2–^ on goethite in the presence of low concentrations of CO_3_^2–^, even when it was also shown that CO_3_^2–^ ions compete with SeO_4_^2–^ for adsorption sites at higher CO_3_^2–^ concentrations. They also observed this with CO_3_^2–^ on SO_4_^2–^, and acetate (CH_3_COO^−^) on SeO_4_^2−^ and SO_4_^2−^. Similar observations were also seen in competitive adsorption studies on Al oxide [54]. They proposed that the coadsorption of H^+^ ions with CO_3_^2−^ ions stabilizes the proton on the surface sites. If the adsorption of another competing anion also involves the coadsorption of H^+^ ions, then the adsorption of the competing anion will be enhanced because the surface is already pre-protonated. Only the anions need to switch places because the protons are already there. Hence, it is entropy driven, which in turn impacts the p*K* values (Eq (1)). Although close proximity and orientation reduces the entropy of reactions, a full understanding of the complex nature of the entropic effect will require an extensive study of the active site environment and its surroundings [55]. Similarly in this study, we propose that the enhanced adsorption strength of Cl^−^ anions in the presence of competing phthalate anions may be due to the enhanced reactivity of the protonated surface sites by the phthalate anions. Once again, only the Cl^−^ and L^2–^ anions need to switch places (in the correct stoichiometric ratios to maintain electroneutrality) because the protons are already there. Thus, the processes involved are both competitive adsorption and enhanced adsorption.

Conversely, the comparatively stronger adsorption of Na^+^ cations on the Sd and Sc surface sites relative to the Sb and Sa sites, seem to get weaker in the presence of phthalate anions. The p*K*_17_ value of 2.8 increases to 5.4 for p*K*_28_, and the p*K*_15_ value of 6.8 increases to 8.8 for p*K*_25_. This suggests that the Na-H exchange is not easy to pursue if the adsorbed H^+^ ion is being stabilized by coadsorbed anions. This observation is based on the proton adsorption data, the assumption that the H:Na stoichiometry is 1:1, and the curve fit with the proton adsorption data shown in Figs 4 and 5. Although we do know that Na^+^ adsorption does occur at high pH values based on the published results by Nanzyo and Watanabe [52], no detailed measurements of Na^+^ adsorption were made in our experiments. Thus, some caution is warranted here with overstating the reasons for the decrease in Na^+^ adsorption strength in the presence of phthalate ions based on the reaction parameters shown in Tables 1 and 2.

The p*K* values reported in Table 2 are similar to the values calculated with the more complicated 2-site CD-MUSIC model for pH > 3, which were –18.24 and –15.37 for phthalate, –8.7 for NO_3_^−^ (same value for both sites), and 0.6 for Na^+^ (same value for both sites) [26]. Their p*K* value for Na^+^ was for an adsorption reaction while ours here are for ion-exchange reactions. Since these p*K* values do not refer to the same Na^+^ reactions, they cannot be compared with each other. The other values reported were, however, for the same ion-exchange reactions. As is common with models based on diffuse layer theories, their model had four additional protonation/deprotonation reactions, which were needed to describe their surface charge reactions on two sites. As with the Na^+^ reactions, their four H^+^ reactions were adsorption (or desorption) reactions, not ion-exchange reactions. A few additional equations and parameters were also included to correlate surface charge with surface potential, which were needed to solve their intrinsic p*K* values.

In our study here, if we ignore all of the Cl^−^ and Na^+^ reactions and focus on only a 2-site adsorption model (or pseudo 2-site model as discussed above) for pH 3 to 14, then we obtain p*K* values of –20.9 and –17.3 with R^2^ = 0.90 for phthalate and R^2^ = 0.98 for H^+^. If we also ignore all of the H^+^ data and fit only the phthalate data in the same pH range, then the p*K* values are –20.9 and –16.8 with R^2^ = 0.93 for phthalate. In the presence of high salt concentrations added, the values shown in Table 1 will also predict H^+^ adsorption quite well if p*K*_14_ is changed to –7.7 and p*K*_10_ is changed to –2.9, where the resultant R^2^ values for the curves generated are then 0.93 for H^+^ with 50 m*M* NaCl added, and 0.82 for H^+^ with 500 m*M* NaCl added. If the p*K*_24_ value in Table 2 is also changed to –7.7 based on these high salt concentration optimization results, then the resultant R^2^ values for the curves generated are 0.99 for H^+^, 0.76 for Cl^−^, and 0.86 for phthalate.

Clearly, a very simple ion-exchange formulation yields very good results, and the inclusion of many competing ions with all of them adhering to strict ion-exchange theories makes the model even more robust, whereby the data can then be predicted to values below pH 3. The ability to include and thus also concurrently predict the H^+^, Cl^−^ and Na^+^ adsorption data with high R^2^ values also exemplifies the strength of this modeling technique. In other words, a strict ion-exchange modeling technique can work very well for us if multiple sites are used as necessary, and it will work even better if all of the competitive ions are also included in the model. Both of these steps have been lacking in traditional ion-exchange models, but they are commonly used in diffuse layer models. We do not verify here (that is, definitively prove, from *verus* for “true”) which is the true nature of the surface complexation reactions involved (such as, strict ion-exchange versus diffuse layer theory). However, we do validate here (that is, demonstrate usefulness) that the much simpler, strict, ion-exchange modeling approach will work very well if constructed properly.

## Conclusions

Phthalate did not require a 4-site model to fit the data, but chloride did. This is probably because the adsorption strength of phthalate is much greater than that of chloride. Weakly adsorbing ions will be more sensitive to very small changes in the surface purity and surface reactivity. The 4-site model for chloride adsorption on goethite agrees with published crystallographic-based discussions of goethite’s reactive surface sites. The pseudo 2-site model for phthalate adsorption on goethite does not contradict this, but instead suggests that three of the sites previously used for describing the adsorption of a weakly held anion can be combined into one site for describing the adsorption of a strongly held anion. The total number of surface sites were conserved for both models in this study.

The curve fits can be done quite well with pure ion-exchange reactions, where the surface charge stays net-neutral at all times. The proposed model was able to concurrently describe the goethite adsorption data of three very different ions (H^+^, Cl^−^ and L^2–^) plus suggest the adsorption envelope expected for a forth ion (Na^+^), which was previously shown in the literature to adsorb on goethite. The goodness-of-fit with the adsorption data collected were very good to excellent.

The success of the proposed model allows us to validate that the proton adsorption data are a result of the 1:1 adsorption stoichiometry of H:Cl and the 2:1 adsorption stoichiometry of H:L on goethite. Even at low pH when Cl^−^ adsorption increased as L^2–^ adsorption decreased, the proposed model tracked these observations and thus clarified why the proton adsorption data were holding steady at the adsorption max value during these competitive anion adsorption reactions.

Another important observation resulting from the optimization of the model was that the retention strength of weakly held Cl^−^ ions increases in the presence of phthalate anions. This anion-anion enhancement effect has been observed before with other anions in other studies, but the model optimization exercise shown here has allowed us to quantify this effect. The retention of Cl^−^ and L^2–^ anions involves both competitive adsorption and enhanced adsorption processes.

There are several different ways of describing surface reactions of divalent ions (see Eqs (6) to (8)), and thus the ion-exchange model introduced here for phthalate plus various competing ions is not definitive. Eq (8) can be dropped as a likely reaction process for low pH data because inclusion of this reaction in the model will yield a changing proton adsorption prediction at low pH (these model results were not discussed here), which contradicts the data collected. Our model here used Eq (7) rather than Eq (6) because it performed better (that is, it fit the data better) and it produces sharper adsorption edges (model results based on Eq (6) were not discussed here). Perhaps additional studies involving spectroscopic analysis, kinetics, and molecular dynamics could help further differentiate the role of Eq (7) from Eq (6). For now, the ion-exchange modeling study presented here confirms that competitive, net charge neutral, strict ion-exchange models will work perfectly well with complex pH-dependent data.

## Supporting information

S1 Table Raw data for Fig 4. Total liquid volume = 35 mL; Solids concentration = 3.43 g/L; Solids specific surface area = 29.5 m^2^/g.
(PDF)

S2 Table Raw data for Fig 5. Total liquid volume = 35 mL; Solids concentration = 3.43 g/L; Solids specific surface area = 29.5 m^2^/g; phthalate (L^2–^) added to all samples = 1 mmol/L.
(PDF)

## Abbreviations

CD-MUSIC: charge-distribution multisite complexation
PZSE: point of zero salt effect
R^2^: coefficient of determination
ZPT: zero point of titration
