# Supplementary material for: Modeling the adsorption of hydrogen, sodium, chloride and phthalate on goethite using a strict charge-neutral ion-exchange theory
Source: PLoS One. 2017 May 2;12(5):e0176743. doi: 10.1371/journal.pone.0176743 (PMC5412999; doi:10.1371/journal.pone.0176743)
Supplement: S1 Table — Total liquid volume = 35 mL; Solids concentration = 3.43 g/L; Solids specific surface area = 29.5 m2/g. (PDF) [file pone.0176743.s001.pdf]

Supporting Information

S1 Table. Raw data for Figure 4.
Total liquid volume = 35 mL; Solids concentration = 3.43 g/L; Solids specific surface area = 29.5 m^2/g.

pH	Na^+ added mmol L^{-1}	Cl^- added mmol L^{-1}	H^+ adsorbed μmol m^{-2}	Cl^- adsorbed μmol m^{-2}
2.5	0.157	2.422	2.0193	
2.51	0.157	1.866	2.4846	
2.8	0.157	1.3	1.8294	2.3148
2.83	0.157	1.187	1.9425	
2.87	0.157	1.017	1.9901	3.4673
2.92	0.157	0.847	1.6108	2.4347
3.15	0.157	0.734	1.701	1.4413
3.43	0.157	0.621	1.4357	1.8424
3.43	0.157	0.536	1.3747	1.7502
3.75	0.157	0.479	1.2929	1.0633
3.76	0.157	0.417	1.2113	1.5147
3.97	0.157	0.292	0.995	
4.01	0.157	0.355	1.1991	1.0279
4.13	0.157	0.294	0.9149	0.8814
4.35	0.157	0.270	0.8162	0.7836
4.49	0.157	0.247	0.687	
4.81	0.157	0.242	0.7721	0.643
5	0.157	0.236	0.6957	
5.21	0.157	0.202	0.429	
5.28	0.157	0.213	0.6181	0.6291
5.49	0.157	0.191	0.4375	
5.6	0.157	0.181	0.2878	
6.04	0.157	0.177	0.3574	0.439
6.41	0.157	0.168	0.1729	0.2774
6.53	0.171	0.168	0.1156	0.1253
6.73	0.195	0.168	-0.1156	0.2521
6.8	0.195	0.168	-0.0107	0.2521
6.8	0.191	0.168	-0.1106	
7.09	0.205	0.168		-0.2297
7.11	0.212	0.168		0.1506
7.19	0.223	0.168		-0.2043
7.23	0.223	0.168	-0.3476	-0.1536
7.27	0.226	0.168	-0.3174	0.0746
7.98	0.24	0.168		-0.179
8.02	0.225	0.168		0.0239
8.48	0.27	0.168		0.2013

8.95	0.27	0.168	-0.3659	
9.43	0.288	0.168	-0.514	
9.55	0.288	0.168	-0.4212	0.2013
9.71	0.308	0.168	-0.5139	-0.3565
9.8	0.35	0.168	-0.6518	0.0239
10.04	0.391	0.168	-0.6437	0.0492
10.39	0.536	0.168	-0.6922	-0.1283
10.66	0.846	0.168	-0.8795	
