# Supplementary material for: Modeling the adsorption of hydrogen, sodium, chloride and phthalate on goethite using a strict charge-neutral ion-exchange theory
Source: PLoS One. 2017 May 2;12(5):e0176743. doi: 10.1371/journal.pone.0176743 (PMC5412999; doi:10.1371/journal.pone.0176743)
Supplement: S2 Table — Total liquid volume = 35 mL; Solids concentration = 3.43 g/L; Solids specific surface area = 29.5 m2/g; phthalate (L2–) added to all samples = 1 mmol/L. (PDF) [file pone.0176743.s002.pdf]

Supporting Information

S2 Table. Raw data for Figure 5.
Total liquid volume = 35 mL; Solids concentration = 3.43 g/L; Solids specific surface area = 29.5 m^2/g; phthalate (L^{2-}) added to all samples = 1 mmol/L.

pH	Na^+ added mmol L^{-1}	Cl^- added mmol L^{-1}	H^+ adsorbed µmol m^{-2}	Cl^- adsorbed µmol m^{-2}	L^{2-} adsorbed µmol m^{-2}
2.33	0.157	3.439	4.289	3.308	1.552
2.52	0.157	2.349	4.521	1.862	1.828
2.67	0.157	1.259	4.298	1.676	1.925
2.87	0.157	0.713	3.773	1.079	1.708
3.06	0.157	0.386	4.385	0.25	2.021
3.11	0.157	0.305	4.423	0.19	2.057
3.15	0.157	0.250	4.194	-0.382	1.888
3.18	0.157	0.168	4.616		2.117
3.79	0.79	0.168	4.376	-0.358	2.213
4.08	0.949	0.168	3.759	0.314	1.864
4.53	1.107	0.168	4.01		1.961
4.74	1.186	0.168	3.553	0.146	1.768
4.92	1.305	0.168	3.449	0.062	1.768
5.22	1.463	0.168	3.396		1.684
5.25	1.543	0.168	3.078		1.155
5.28	1.543	0.168	3.196	-0.526	1.48
5.32	1.543	0.168	2.982	-0.274	1.071
5.42	1.582	0.168	3.166	-0.19	1.443
5.43	1.582	0.168	2.958	0.566	1.01
5.55	1.701	0.168	2.468	0.314	0.866
5.59	1.701	0.168	2.692	-0.526	1.071
5.63	1.701	0.168	2.63	-0.106	
6.08	1.859	0.168	2.196	0.398	1.203
6.11	1.859	0.168	2.104	-0.022	
6.37	1.938	0.168	1.849	0.062	0.986
6.53	1.978	0.168	1.599	-0.106	0.938
6.84	2.041	0.168	1.023	-0.106	0.445
7.04	2.097	0.168	0.53	0.062	0.277
7.51	2.136	0.168	0.176	-0.274	0
8.35	2.176	0.168	-0.213	-0.022	0.409
9.09	2.216	0.168	-0.414	-0.022	0.156
10.39	2.414	0.168	-0.697		
10.76	3.324	0.168		-0.442	0.108
